# Sphenoid Mucocele Complicated With Partial Medial Pontine Syndrome

**DOI:** 10.1002/oto2.70159

**Published:** 2025-09-02

**Authors:** Yu‐Han Wang, Ya‐Fang Chen, Ting‐Hua Yang, Chih‐Feng Lin

**Affiliations:** ^1^ Department of Medical Education National Taiwan University Hospital Taipei Taiwan; ^2^ Department of Medical Imaging National Taiwan University Hospital Taipei Taiwan; ^3^ Department of Otolaryngology, Head and Neck Surgery National Taiwan University Hospital Taipei Taiwan; ^4^ Institute of Medical Device and Imaging National Taiwan University Taipei Taiwan

**Keywords:** brainstem Infarction, partial medial pontine syndrome, sphenoid mucocele

Sphenoid mucoceles are rare, accounting for less than 2% of paranasal sinus lesions.^1^ They result from chronic sinus ostium blockage, leading to mucus buildup, sinus expansion, and possible bone erosion. Their central location puts nearby structures at risk, including the optic nerves and the basilar artery.

Ischemic stroke caused by sphenoid mucoceles is extremely rare, and may occur due to local inflammation or direct compression of nearby vessels.^2^, ^3^ The ventral pons, supplied by paramedian branches of the basilar artery, is especially vulnerable. Infarction in this area can cause partial medial pontine syndrome, characterized by contralateral hemiparesis and central facial weakness, while preserving cranial nerve nuclei.^4^ This case highlights the importance of early diagnosis and timely surgery when vascular compromise is suspected.

## Case Report

A 74‐year‐old man with hypertension and chronic rhinosinusitis, previously treated with Luc's operation 50 years ago and bilateral functional endoscopic sinus surgery 7 years ago, presented with a 10‐day history of progressive dysarthria and brief, cough‐induced headaches. Five days prior to admission, he developed right central facial weakness, right hemiparesis, right‐sided hypoesthesia, and dysmetria.

**Figure 1:**
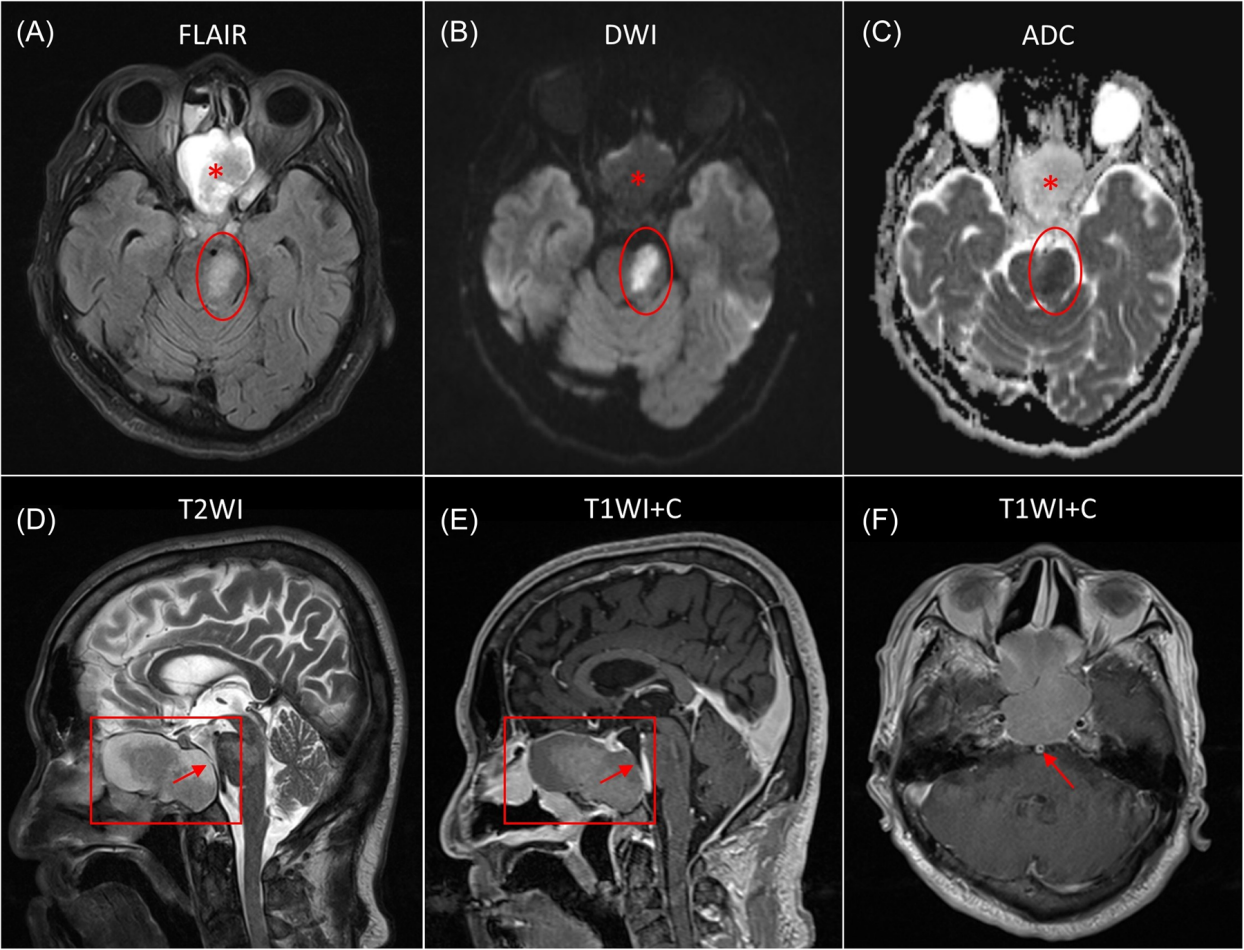
MRI: (A) Left pontine infarct and sphenoid mucocele (hyperintensity); (B) DWI shows restricted diffusion; (C) ADC map shows facilitated diffusion; (D, E) Sagittal images reveal expansile lesion and mucosal thinning; (F) Wall enhancement of basilar artery.

Upon arrival, his National Institutes of Health Stroke Scale (NIHSS) score was 8. Non‐contrast head computed tomography showed a soft tissue mass in the posterior ethmoid and sphenoid sinuses, with possible skull base erosion and suspected left pontine hypodensity. Magnetic resonance imaging (MRI) axial T2‐weighted fluid‐attenuated inversion recovery (FLAIR), diffusion‐weighted imaging (DWI), and apparent diffusion coefficient (ADC) map images confirmed a recent left pontine infarct, and revealed a 5.4 × 4.2 × 3.6 cm expansile sphenoid sinus lesion (Figure 1A–C). Sagittal T2‐weighted imaging (T2WI) and postcontrast sagittal T1‐weighted imaging (T1WI) demonstrate a predominantly hyperintense expansile lesion within the sphenoid sinus, reflecting mucoid content with internal signal heterogeneity, and focal mucosal thinning at the posterior wall of the sphenoid sinus (Figure 1D,E). Enhancement of the adjacent basilar artery wall were observed (Figure 1F), suggesting localized vascular inflammation.

**Figure 2:**
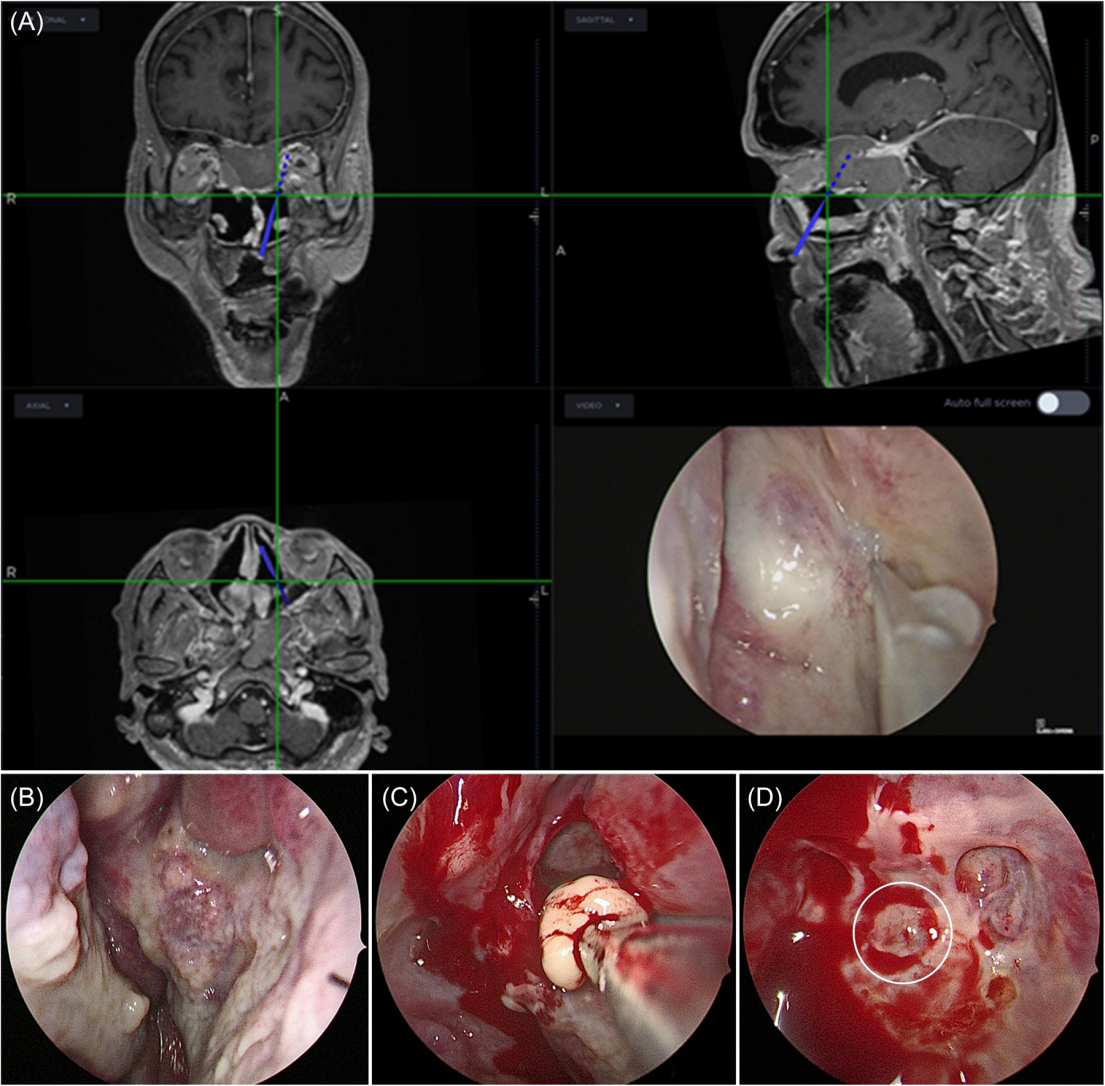
Intraoperative endoscopic views of the sphenoid sinus mucocele: (A) navigation to delineate boundaries; (B) large expansile mucocele; (C) evacuation of mucoid material; (D) posterior wall thinning (circle).

Due to suspected mucocele‐related infarction, he underwent emergent bilateral endoscopic sinus surgery under image guidance (Figure 2A). A wide posterior septectomy and bilateral sphenoidotomies were performed to evacuate thick mucoid and mucopurulent material (Figure 2B,C). The posterior sphenoid wall exhibited a focal area of thinning, but no cerebrospinal fluid leakage, or skull base defect was noted (Figure 2D). The patient tolerated the procedure well.

By postoperative day one, his NIHSS score improved to 5, with enhanced right upper limb strength, sensation, and coordination. Stroke workup showed no additional risk factors. He remained neurologically improved and was transferred to the rehabilitation ward one week later.

This case report was exempted by the Institutional Review Board of National Taiwan University Hospital (registration number: 202506139W).

## Discussion

Sphenoid mucoceles are rare but potentially serious lesions caused by obstruction of the sinus ostium, leading to mucus accumulation. This may result from chronic rhinosinusitis, postoperative scarring, polyps, trauma, or tumors. As the mucocele enlarges, it can erode bone and compress nearby structures like the internal carotid arteries (ICAs), optic nerves, and cranial nerves III to VI. Patients typically present with headache, visual changes, or cranial nerve deficits. In rare cases, complications such as ischemic stroke may occur.

Sphenoid mucoceles can cause stroke through either inflammation or mechanical compression, with inflammation being more common. Infected or ruptured mucoceles may induce localized inflammation that spreads to adjacent vascular endothelium, resulting in endothelial injury, vasospasm, or thrombosis.^2^ Similar processes have been observed in sphenoid sinusitis without mucocele formation, indicating that paranasal inflammation alone can affect neighboring vessels.^5^

Mechanical compression is a plausible mechanism in large mucoceles eroding sinus walls near major arteries, potentially causing luminal narrowing and altered flow. In rare instances, giant mucoceles have compressed both internal carotid arteries, leading to cerebral ischemia.^3^ Imaging in this case showed no basilar artery compression but revealed focal wall enhancement adjacent to the mucocele, indicating inflammation. The patient presented with contralateral hemiparesis, dysarthria, and central facial palsy, features consistent with partial medial pontine infarction. MRI confirmed infarction in the paramedian branches of the basilar artery near the inflamed sinus wall, supporting a causal association.

This case underscores the potential role of sphenoid mucoceles in precipitating pontine infarction through localized inflammatory mechanisms. Recognition of this association is essential in patients presenting with concurrent sinus disease and brainstem ischemia, even in the absence of clear radiographic vascular compromise. Timely diagnosis and surgical management can mitigate serious complications and facilitate neurological recovery.

## Author Contributions

**Yu‐Han Wang**: acquired the data and drafted the manuscript; **Ya‐Fang Chen**: acquired the data and revised the manuscript; **Ting‐Hua Yang**: analyzed the data and revised the manuscript; **Chih‐Feng Lin**: designed the study and approved the manuscript.

## Disclosures

### Competing interests

None.

### Funding source

This study was supported by Taiwan National Science and Technology Council grant 114‐2320‐B‐002‐024.
